# Preparation, characterization, *in vitro* drug release and anti-inflammatory of thymoquinone-loaded chitosan nanocomposite

**DOI:** 10.1016/j.jsps.2022.02.002

**Published:** 2022-02-07

**Authors:** Mothanna Sadiq Al-Qubaisi, Ashwaq Shakir Al-Abboodi, Fatah H. Alhassan, Samer Hussein-Al-Ali, Moayad Husein Flaifel, Eltayeb E.M. Eid, Hussah Abdullah Alshwyeh, Mohd Zobir Hussein, Sulaiman Mohammed Alnasser, Mohammed Ibrahim Saeed, Abdullah Rasedee, Wisam Nabeel Ibrahim

**Affiliations:** a Institute of Bioscience, Universiti Putra Malaysia, 43400 UPM Serdang, Selangor, Malaysia; b Basic Science Branch, Faculty of Dentistry, University of Al-Qadisiyah, Al-Diwaniyah, Iraq; c Department of Applied Chemistry and Technology, College of Science and Arts, Alkamel University of Jeddah, Jeddah 21589, Saudi Arabia; d Faculty of Pharmacy, Isra University, Amman 11622, Jordan; e Department of Physics, College of Science, Imam Abdulrahman Bin Faisal University, Dammam 31441, Saudi Arabia; f Basic & Applied Scientific Research Center, College of Science, Imam Abdulrahman Bin Faisal University, Dammam 31441, Saudi Arabia; g Department of Pharmaceutical Chemistry and Pharmacognosy, Unaizah College of Pharmacy, Qassim University, Saudi Arabia; h Department of Biology, College of Science, Imam Abdulrahman Bin Faisal University, Dammam 31441, Saudi Arabia; i Department of Chemistry, Faculty of Science, Universiti Putra Malaysia, 43400 UPM Serdang, Selangor, Malaysia; j Department of Pharmacology and Toxicology, Unaizah College of Pharmacy, Qassim University, Saudi Arabia; k Faculty of Medical Laboratory Sciences, National Ribat University, Khartoum 11111, Sudan; l Department of Veterinary Pathology and Microbiology, Faculty of Veterinary Medicine, Universiti Putra Malaysia, 43400 UPM Serdang, Selangor, Malaysia; m Department of Biomedical Science, College of Health Sciences, QU Health, Qatar University, Doha, Qatar; n Biomedical and Pharmaceutical Research Unit, QU Health, Qatar University, Doha, Qatar

**Keywords:** Thymoquinone, Nanocomposite, High-pressure homogenizer, Drug release, RAW 264.7 cells, Inflammatory mediators

## Abstract

In this study, we formulated Thymoquinone-loaded nanocomposites (TQ-NCs) using high-pressure homogenizer without sodium tripolyphosphate. The TQ-NCs were characterized and their anti-inflammatory determined by the response of the LPS-stimulated macrophage RAW 264.7 cells in the production of nitric oxide, prostaglandin E2, tumor necrosis factor-α, interleukin-6, and interleukin-1β. The physicochemical properties of TQ-NC were determined using different machines. TQ was fully incorporated in the highly thermal stable nanoparticles. The nanoparticles showed rapid release of TQ in the acidic medium of the gastric juice. In medium of pH 6.8, TQ-NC exhibited sustained release of TQ over a period of 100 h. The results suggest that TQ-NC nanoparticles have potential application as parenterally administered therapeutic compound. TQ-NC effectively reduce production of inflammatory cytokines by the LPS-stimulated RAW 264.7 cells, indicating that they have anti-inflammatory properties. In conclusion, TQ-NC nanoparticles have the characteristics of efficient carrier for TQ and an effective anti-inflammatory therapeutic compound.

## 1 Introduction

The full potential of chitosan as a biomaterial is yet to be realized. Chitosan has been shown to induce the proliferation of immune cells and increase chemokine production in wounds (Sarmento and das Neves, 2012, Dutta, 2015, Ahmed and Ikram, 2017). This compound has also been shown to increase the infiltration of leukocytes and macrophages in wounds and enhancing healing (Inui et al., 1995, Dutta, 2015). Chitosan exerts its effect by increasing production of growth factors such as colony stimulating factor (CSF) and platelet-derived growth factor (PDGF) (Brown et al., 1989, Inui et al., 1995, Ueno et al., 2001), decreasing interleukin-4 (IL-4), interleukin-5 (IL-5), interleukin-13 (IL-13), and tumor necrosis factor-α (TNF-α) (Chung et al., 2012) production by basophils and pro-inflammatory markers by osteoblasts (Kim et al., 2011). The compound has also been reported to decrease nitric oxide production by macrophages *in vitro* (Yang et al., 2010).

*Nigella sativa*, a genus in the family of Ranunculaceae (Ravindran, 2017), was traditionally used for treatment of coughs, colds, sore throats, and wounds (Mouhajir et al., 2001, Kunnumakkara et al., 2009, Rahmatullah et al., 2010). The *N. sativa* oil extract is used to treat upper respiratory tractand ear infections and wounds (El-Magboub, 2011, Saad, 2015, Hussain and Hussain, 2016). There are experimental evidences that suggest thymoquinone (TQ) extracted from *N. sativa* possess a variety of bioactivities including immunomodulatory (Majdalawieh and Fayyad, 2015), anti-inflammatory (El Gazzar et al., 2006, Ragheb et al., 2009), antiviral (Forouzanfar et al., 2014), antimicrobial (Forouzanfar et al., 2014), antioxidant (Nagi et al., 1999), and anticancer activities (H El-Far, 2015). In spite the therapeutic effects, TQ is poorly water-soluble, which compromises its potential as a therapeutic compound. There are several carriers that can be used to increase the solubility of TQ (Al-Qubaisi et al., 2019). Among these carriers is chitosan with characteristic polymeric cavity that could accommodated loaded TQ. The chitosan nanoparticles are usually prepared by ionotropic gelation of water-soluble chitosan with sodium tripolyphosphate. Chitosan is positively charged and can form a complex with the negatively charge sodium tripolyphosphate (Yang et al., 2009). However, sodium tripolyphosphate is detrimental to human health and the environment. The compound has been linked to kidney disease, skin inflammation, intestine irritation, heart problems, and even premature death (Madsen et al., 2001; Uribarri, 2007, Ritz et al., 2012, Calvo and Uribarri, 2013, Kalantar-Zadeh, 2013). Therefore, as an alternative, we used the water-soluble mushroom chitosan, without the use of sodium tripolyphosphate, in the preparation of TQ-loaded nanocomposite for the determination of anti-inflammmatory effects. The inflammation-modulation effect of the nanocomposite was determined *in vitro* on the lipopolysaccharide-stimulated RAW 264.7 cells.

## 2 Materials and methods

### 2.1 Chemicals and reagents

Trypsin/Ethylenediaminetetraacetic acid (EDTA) solution was purchased from Invitrogen (Carlsbad CA, USA). Dimethylsulfoxide (DMSO), phosphate-buffered saline (PBS), 3-(4,5-dimethylthiazol-2-yl)-2,5-diphenyltetrazolium bromide (MTT), and Dulbecco’s modified Eagle’s medium (DMEM) were purchased from Sigma Chemical Company (PerthWestern, Australia). Thymoquinone (TQ) was purchased from sigma Aldrich (St. Louis, MO, USA). Water soluble mushroom chitosan was provided by Xi’an Surnature Biological Technology Co. Ltd, China. The water-soluble mushroom chitosan specifications were as follows: molecular weight is 161.16 g mol^−1^, deacetylation DD is 95.0%, ash content is ≤1.0%, and viscosity is 11 CPS.

### 2.2 Preparation of thymoquinone-loaded chitosan nanocomposite

Thymoquinone-loaded chitosan nanocomposite (TQ-NC) nanoparticles were prepared by homogenizing chitosan (900 mg in 10 mL) with thymoquinone (100 mg in 90 mL) using two methods. The first method involved homogenization of solutions in an Ultra-Turrax T25 (IKA, Staufen, Germany) for 1 min at 4000, 8000, 12,000, 16,000, 20,000 and 24,000 rpm. The second method used a bench-top high-pressure homogenizer (Stansted Fluid Power, Ltd., Essex, UK) at 800 bar for 5 cycles to produce the nanocomposite. To avoid degradation of TQ, after each homogenization cycle, the mixture was cooled to <25 °C using an ice bath. The nanoparticles were dried by spray drying at 25 °C and then ground to obtain powder.

### 2.3 Physicochemical properties of nanoparticles

#### 2.3.1 Scanning electron microscopy

Scanning electron microscopy (SEM) [Model LEO 1450VP (LEO Electron Microscopy Ltd Cambridge, UK)] with an accelerating voltage of 30 kV were used to determine the morphology of TQ-NC nanoparticles. The samples were degassed in an evacuated heated chamber at 100 °C overnight. Prior to SEM scanning, dried samples were spread over double-sided conductive tape and adhered to the specimen stub.

#### 2.3.2 Transmission electron microscopy

Transmission electron microscopy (TEM) (Model CM12; Philips, Eindhoven, The Netherlands) with an accelerating voltage of 120 kV and a maximum magnification limit of 660 k times, was used to determine the homogeneity of the TQ-NC nanoparticles. The nanoparticles sample was homogeneously dispersed in deionized water in an ultrasonic bath and dropped onto copper grids placed on a filter paper and then dried at room temperature.

#### 2.3.3 X-ray diffraction

The powder X-ray diffraction (XRD) patterns of TQ, water-soluble mushroom chitosan, and powdered TQ-NC nanoparticles were recorded with a Shimadzu XRD-6000 instrument (Shimadzu Corporation, Kyoto, Japan) in the range of 10–80° using CuKα as a radiation source (λ = 1.5418 Å) generated at 30 kV and 30 mA.

#### 2.3.4 Fourier transform infrared

Fourier transform infrared FTIR spectra for TQ, water-soluble mushroom chitosan, and powdered TQ-NC nanoparticles were recorded over the range of 400–4000 cm^−1^ on a Thermo Nicolet Nexus, Smart Orbit spectrometer (Shelton, USA) using 1% in 200 mg spectroscopic-grade potassium bromide (KBr) under 10 tons of pressure.

#### 2.3.5 Thermogravimetric analysis

The thermal strength of TQ, water-soluble mushroom chitosan, and powdered nanocomposite was investigated using the Mettler Toledo TG-SDTA apparatus (Pt crucibles, Pt/Pt–Rh thermocouple) (Switzerland), with a purge gas (nitrogen) flow rate of 30 mL min^−1^ and heating rate of 10 °C min^−1^, from room temperature to 1000 °C.

#### 2.3.6 Differential scanning calorimetry

Differential scanning calorimetry (DSC) (Model Mettler Toledo 821^e^) was used to determine sample melting point (T_m_) at the temperature range of 23–599 °C using nitrogen gas pressure and a heating rate of 10 °C/min.

#### 2.3.7 Hydrodynamic size and zeta potential

The hydrodynamic size and zeta potential of the TQ-NC nanoparticle suspension (1 μg nanoparticles dispersed in 1 mL of ultra-deionized water) were determined using the ZetaSizer Nano ZS (Malvern Instruments Ltd Malvern, UK) with dynamic light scattering.

#### 2.3.8 Drug loading and entrapment efficiency

The loading and entrapment efficiency of TQ were determined by UV–Vis spectrophotometer (Model Perkin Elmer Lambda 35). A calibration curve for a standard was constructed and the drug-loading and drug-entrapment efficiency using the following equations:

(1) $$\text{Entrapment efficiency}\;\left(\%\right)\;=\frac{\text{Initial drug amount}\;\left(\text{mg}\right)-\;\text{Unentrapped drug}\;\left(\text{mg}\right)}{\text{Initial drug amount}\;\left(\text{mg}\right)}\times 100$$

(2) $$Drug\;loading\;\left(\%\right)=\frac{Initial\;drug\;amount\;\left(mg\right)-Unentrapped\;drug\;\left(mg\right)}{Amount\;ofTQ-NC\;nanoparticles\;\left(mg\right)}\times 100$$

#### 2.3.9 Drug release

*In vitro* TQ release characteristics from the nanoparticles were determined by adding 1.0 mg of powdered TQ-NC to 3 mL of either artificial gastric juice (AGJ) [pH 1.2, phosphate buffered saline solution (PBS) containing 0.1 μM HCl and 3.2% pepsin], or artificial intestinal juice (AIJ) [pH 6.8, PBS containing 3 μM phospholipid and 10 μM bile salt]. The cumulative amount of TQ released into the solution was measured at preset time intervals using a UV–Vis spectrophotometer (Model Perkin Elmer Lambda 35) at λ_max_ = 254 nm. The KinetDS 3.0 software (Mendyk et al., 2012) was used to determine the TQ-release profile from the nanoparticles (Mendyk et al., 2012). The results from the drug release determination were fitted to kinetic models of zero-order (3) and first-order (4), second-order (5), Higuchi (6), KorsmeyerPeppas (7), Weibull (8), Hixson-Crowell (9), Michaelis–Menten (10), and Hill (11). The best-fit determined the model that best described the drug release curve, mainly by the empirical R^2^ R^2^_emp_ (16), Akaike information criterion AIK (17), Bayesian information criterion BIC (18) and root-mean-squared error RMSE (19). The dissolution efficiency (DE) (12) and mean dissolution time MDT (13) were also computed using the KinetDS 3.0 software (Patel et al., 2008).

(3) $$Q=k.t+Q_{0}$$

(4) $$\frac{1}{Q}=k.t+\frac{1}{Q_{0}}$$

(5) $$\frac{1}{Q^{2}}=k.t+\frac{1}{Q_{0}^{2}}$$

(6) $$Q=k.\sqrt{t}$$

(7) $$Q=k.t^{n}$$

(8) $$Q=100.\left[1-exp\left(\frac{-{\left(t-T_{\mathrm{LAG}}\right)}^{b}}{a}\right)\right]$$

(9) $$Q^{\frac{1}{3}}=k.\left(t-T_{\mathrm{LAG}}\right)+Q_{0}^{\frac{1}{3}}$$

(10) $$Q=\frac{Q_{\max}.t}{k+t}$$

(11) $$Q=\frac{Q_{\max}.t^{n}}{k^{n}+t^{n}}$$

(12) $$\mathrm{DE}=\frac{\int_{0}^{t}Qdt}{Q_{\max}.t}*100$$

(13) $$\mathrm{MTD}=\frac{\sum_{j=1}^{n}t_{j}^{\mathrm{AV}}*\Delta Q_{j}}{\sum_{j=1}^{n}\Delta Q_{j}}$$

(14) $$\Delta Q=Q_{\left(t\right)}-Q_{\left(t-1\right)}$$

(15) $$t_{j}^{\mathrm{AV}}=\left(t_{i}+t_{i-1}\right)/2$$

(16) $$R_{\mathrm{emp}}^{2}=1-\frac{\sum_{i=1}^{N}{\left(y_{i}-{\hat{y}}_{i}\right)}^{2}}{\sum_{i=1}^{N}{\left(y_{i}-y_{\mathrm{AV}}\right)}^{2}}$$

(17) $$\mathrm{AIC}=2k+N.\left[In\left(\sum_{i=1}^{N}{\left(y_{i}-{\hat{y}}_{i}\right)}^{2}\right)\right]$$

(18) $$\mathrm{BIC}=N.In\left(\sum_{i=1}^{N}{\left(y_{i}-{\hat{y}}_{i}\right)}^{2}\right)+k.In\left(N\right)$$

(19) $$\mathrm{RMSE}=\sqrt{\frac{\left(\sum_{i=1}^{N}{\left(y_{i}-{\hat{y}}_{i}\right)}^{2}\right)}{N}}$$

Q = amount (%) of drug substance released at the time t, Q_0_ = start value of Q, Q_max_ = maximum value of Q (100%), T = time, k, a, b = constant, T_LAG_ = lag time, y_i_ = observed value, ŷ_i_ = model-predicted value, y_AV_ = average output value (Patel et al., 2008).

### 2.4 Cell culture

Virus-negative mouse macrophages (RAW 264.7) cells (passage number 8–13) grown as an adherent monolayer of tightly knit epithelial cells were purchased from the American Type Culture Collection (ATCC; Rockville, MD, USA). The cells in DMEM (Sigma Aldrich, USA) supplemented with 10% fetal bovine serum (FBS) and 1% penicillin (100 U/mL) (Isocillin, Aventis, Germany) were cultured in an incubator at 37 °C under 5% CO_2_.

### 2.5 Cytotoxicity

#### 2.5.1 3-(4,5-dimethylthiazol-2-yl)-2,5-diphenyltetrazolium bromide assay

Cytoxicity of thymoquinone is likely related to the concentration added to cells. We should determine the concentration (of our compound) which may result in lowest toxicity as possible for safety purpose (Al-Qubaisi et al., 2019). The wells of a 96-well tissue culture plate were seeded with 200 μL of 1 × 10^5^ cells/mL RAW 264.7 cells in suspension. The plates were incubated for two days to ensure attachment at 80–90% confluency. The medium was aspirated and replaced with either 200 μL of 1.563, 3.125, 6.25, 12.5, or 25 μg/mL TQ encapsulated in water soluble mushroom chitosan or dissolved in DMSO, or pure water-soluble mushroom chitosan. The last row was left as an untreated control. The plates were incubated at 37 °C for 24 h under 5% CO_2_. After incubation, the medium was aspirated and the cells washed thrice with PBS buffer and 200 µL fresh medium added to each well. 20 μL MTT solution was added and made to a total volume of 200 μL with medium, gently mixed, and incubated at37 °C for 4 to 6 h under 5% CO_2_. The MTT-containing medium was then carefully removed and replaced with 200 μL DMSO/well to dissolve the formazin crystals. The plates were read on an automated spectrophotometric EL 340 multiplate reader (Bio-Tek Instruments Inc., USA) at 570 nm. Following Mosmann’s method (Mosmann, 1983), the viability percentage is calculated by:

(20) $$\frac{{\mathrm{OD}}_{\mathrm{treated}\;\mathrm{well}}}{{\mathrm{OD}}_{\mathrm{nontreated}\;\mathrm{well}}}\times 100$$

OD is optical density

In addition, the cells were treated with serial concentrations of above mentioned compounds for 24 h then challenged with lipopolysaccharide LPS (100 ng/ml) for two hours.

### 2.6 Anti-inflammatory activity

#### 2.6.1 LPS-activated RAW 264.7 cells

200 µL of suspension containing 2 × 10^5^ RAW 264.7 cells/ mL were added to each well of 6-well plates and incubated for 24 h. Confluent cells were then pre-treated with 0, 0.125, 0.25, 0.5, 1.0 and 2.0 µg/mL TQ dissolved in DMSO or encapsulated in water soluble mushroom chitosan for 24 h. 100 ng/mL LPS was added to cells and the plate incubated for 2 h before the determination of pro-inflammatory markers production.

#### 2.6.2 Nitric oxide (NO)

The production of nitric oxide by the compounds-pretreated LPS-activated cells was determined by the nitrite concentration in the supernatant according to the Griess reaction (Kim et al., 1999, Ranneh et al., 2016). The Griess reagent containing 0.1 % N-1-[naphthyl] ethylenediamine–dihydrochloride (NEDD), 1% sulphanilamide, and 5% phosphoric acid was vortexed to evenly mix. 100 µL of cell culture supernatants were incubated (1:1) with Griess solution (Sigma-Aldrich, St. Louis, MO) for 20 min at room temperature. The optical densities were measured at 550 nm and nitric oxide concentration was determined using a standard curve. Resveratrol (5 μg/mL), an inhibitor of nitric oxide production, was used as the positive control.

#### 2.6.3 Prostaglandin E2 (PGE2)

The concentration of PGE2 produced by the compounds-pretreated LPS-activated RAW 264.7 cells was determined using an immunoassay kit (R&D system, USA). A 3-fold dilution of the supernatant from the wells containing the cells was made. Approximately 150 µL of the diluted supernatant or standard was added to the designated wells. Exactly 50 µL of primary antibody solution was added to the wells containing the samples or the standard. The plates were sealed and agitated on the shaker for one hour at room temperature. Then 50 µL of PGE2 conjugate was added to each well and the plates sealed and agitated for 2 h. Each well was aspirated and washed thrice with wash buffer. 200 µL of substrate solution was added to each well and the plate agitated at room temperature in the dark for 30 min. Finally, 100 µL of stop solution was added to each well. Appearance of yellow color indicates end of reaction. The optical density of each was determined at 450 nm using an ELISA plate reader. Resveratrol (5 μg/mL), an inhibitor of PGE2 production, was used as the positive control.

#### 2.6.4 Interleukin-1β (IL-1β), Interleukin-6 (IL-6), and tumor necrosis factor-α (TNF-α) proteins

The medium in the wells containing the compounds-pretreated LPS-activated RAW 264.7 cells was centrifuged at 2000*g* (Eppendorf 5810R centrifuge) for 5 min and the supernatant stored in −20 °C. The TNF-α, IL-1β and IL-6 concentrations in the supernatants were determined using ELISA kits (R&D system, USA). Briefly, 100 µLin PBS of capture antibody was added to selected wells of a 96-well plate. The plate was sealed with aluminium foil and incubated at room temperature overnight with agitation, the medium removed, and the plate washed at least thrice with wash buffer. The reaction was then blocked with the addition of 300 µL blocking buffer to each well and the plate allowed to stand for 1 h at room temperature. After aspiration of the medium and washing at least thrice with wash buffer, 100 µL of either samples or standard were added to the designated wells, the plates covered with aluminium foil and incubated at room temperature for 2 h. All incubations were done with moderate agitation to maximize interactions. The medium was aspirated and the wells washed as described previously. 100 µL of biotinylated detection antibody was added, the plate covered with aluminium foil and reincubated at room temperature for 2 h before again aspirating medium and washing. 100 µL of streptavidin-HRP was added to each well and the plates incubated at room temperature with agitation for 20 min away from direct light. After aspiration and washing, 100 µL of substrate solution was added to each well, the plate covered with aluminium foil and incubated in the dark for 30 min. Finally, 50 µL of stop solution was added to each well. The optical density of each well was immediately determined at 450 nm. Standard curves were prepared for the estimation of cytokine concentrations.

### 2.7 Statistical analysis

All experiments were done in triplicates unless otherwise indicated. The data were expressed as means ± standard deviation. All statistical analyses were performed using the Minitab statistical software (Minitab Inc., State College, PA). The significance of treatment effects was determined using one-way analysis of variance (ANOVA) followed by the Dunnett's comparison tests. Significance among means was determined at p < 0.05.

## 3 Results

### 3.1 Characterization

#### 3.1.1 Morphological analysis of nanoparticles

##### 3.1.1.1 Scanning electron microscopy SEM

**Fig. 1:**
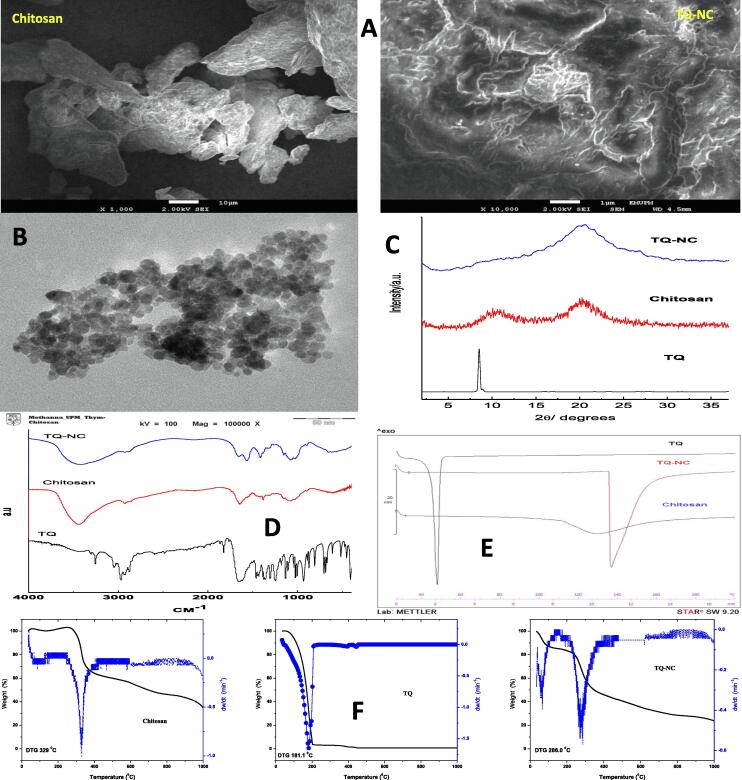
Characterization of chitosan, thymoquinone (TQ), and thymoquinone-loaded chitosan nanocomposite (TQ-NC) by: (A) SEM micrographs of chitosan and TQ-NC. (B) Transmission electron micrograph of TQ-NC. (C) X-ray diffraction (XRD) pattern of the samples. (D) Fourier transform infrared spectrum of samples. (E) Differential scanning calorimetric curves for the samples. (F) Thermogravimetric analysis-differential thermogravimetry thermograms for the samples.

The SEM images show that the blank chitosan had a wide range of particle size and shape (Fig. 1A). The TQ-NC nanoparticles, however, were relatively distorted in shape and had rough surface and were aggregated. No discrete nanoparticle was observed.

##### 3.1.1.2 Transmission electron microscopy TEM

The TEM image showed the lateral dimension of the TQ-NC nanoparticles had an average diameter of 12 nm (Fig. 1B).

#### 3.1.2 X-ray powder diffraction

X-ray powder diffraction (XRD) analysis for TQ, chitosan, and TQ-NC nanoparticle for the 2θ range 2.2–40.0° is shown in Fig. 1C. The characteristic diffraction pattern of TQ was at 10.4 Å, while the chitosan shows diffraction patterns at 7.8 and 20.4 Å. The XRD patterns suggest that TQ-NC nanoparticle either formed a molecular or amorphous dispersion within the chitosan matrix.

#### 3.1.3 Infrared absorption bands

The Fourier transform infrared FT-IR spectra of TQ, chitosan, and TQ-NC nanoparticles are presented in Fig. 1D. The broad band at 3448 cm^−1^ in chitosan spectra is attributed to N-H, O-H stretching vibrations via hydrogen bonds, and the weak band at 2924 cm^−1^ is ascribed to C—H stretch of chitosan. The characteristic amide band and N—H bend vibration of chitosan are clearly viewed at 1637 cm^−1^ and 1524 cm^−1^, respectively. The band near 1079 cm^−1^ is attributed to the stretching of CO in the COH, COC and CH_2_OH ring. The bands for TQ at 2968, 2923 and 1430 cm^−1^ are attributed to the stretching of asymmetric CH_3_. The bands at 2878 and 1360 cm^−1^ are assigned to the symmetric stretching modes of the methyl groups. The band at 1646 cm^−1^represents C=O stretching. The out-plane C—H bending modes appeared at 1249 cm^−1^. The bands located at 672–612 cm^−1^ are due to the ring bending modes. The FTIR for TQ-NC nanoparticles shows that the functional groups of TQ and chitosan with some shift. The bands at 2926, 1653, 1075, and 619 cm^−1^ are associated with chitosan, whereas the bands at 1653, 1413, 1343, 1262, 1154, 1028, 899, and 653 cm^−1^ are associated with TQ. The interaction between TQ with chitosan in the TQ-NC nanoparticle is presented in Fig. 1D.

#### 3.1.4 Differential scanning calorimetry

Differential scanning calorimetry (DSC) is a mean for the determination of the physical and the energetic properties of a compound or formulation by determining heat loss or gain, as a function of the temperature (Reading and Hourston, 2006, Höhne et al., 2013). Fig. 1E represents the DSC results of TQ, chitosan, and TQ-NC nanoparticle. The DSC thermogram for TQ shows an endothermic peak at 46.25 °C, which corresponds to the melting point of the compound. Pure chitosan had a melting point of 129.21 °C (Table 1). The endothermic peak for chitosan and TQ-NC was at 74 °C (Table 1), which possibly represent loss of water. The thermograms of the TQ-NC nanoparticles showed sharp endothermic peak in the temperature range investigated when a compared to pure chitosan. The endothermic peak for TQ-NC nanoparticles at 135.43 °C indicates a strong interaction between TQ and chitosan. This shows that TQ-NC nanoparticles are not just simple chemical mixture but a stable complexation of TQ and chitosan. The enthalpy, melting point and onset point of TQ, chitosan and TQ-NC are shown in Table 1.

**Table 1:** The differential scanning calorimetric analysis of thymoquinone (TQ), chitosan and TQ-loaded chitosan nanocomposite.

Sample	Onset (°C)	Melting point (°C)	Enthalpy (J/g)
Thymoquinone	44.67	46.25	101.83
Chitosan	111.83	129.21	118.00
Thymoquinone-loaded chitosan nanocomposite	135.25	135.43	335.36

#### 3.1.5 Thermogravimetric analysis

The thermal gravimetric analysis (TGA) of chitosan, TQ and TQ-NC nanoparticles are shown in Fig. 1F. There was a sharp weight loss between 77.6 and 210 °C of the thermgram that corresponds the decomposition of TQ. For TQ, only one peak is seen at 181.1 °C. Since TQ is a hydrophobic compound, a significant amount of water is not observed in the other stages. For chitosan, in the first stage there was a loss of mass at 62.6 °C, which is attributed to loss of water, whereas the 329 °C in the second stage corresponds to the thermal decomposition of chitosan. The decomposition of chitosan began at 245.1 °C and ended at 420.0 °C, with total weight loss of 37.1%. It appears that the amount water molecules in chitosan are limited by its two polar groups, hydroxyl and amine. In the TQ-NC nanoparticles, the mass loss due to water loss, was observed in the first phase at 65.1 °C. The second stage at 286.0 °C corresponds to the thermal and oxidative decomposition of chitosan and the vaporization and elimination of volatile contents. The third stage at 455.0 °C represents the thermal decomposition of the sample. Decomposition of TQ in TQ-NC nanoparticles occurred at 240 °C. This suggests that the thermal stability of TQ-NC nanoparticles was enhanced by the interaction between the C=O amide functional group of TQ and the amine group of chitosan.

#### 3.1.6 Hydrodynamic size and zeta potential

The TQ-NC nanoparticles had hydrodynamic diameters of 186.3 ± 25.9 nm, polydispersity index (PDI) of 0.367 ± 0.017, and zeta potential of 49 ± 21 mV.

#### 3.1.7 Drug release, loading and encapsulation efficiency

**Fig. 2:**
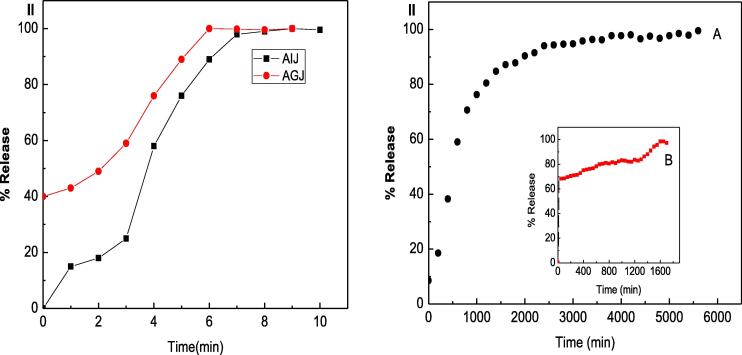
(I) Release profiles of thymoquinone (TQ) from physical mixture of thymoquinone with chitosan in artificial intestinal juice (AIJ) and artificial gastric juice (AGJ). (II) Release profiles of TQ from TQ-loaded chitosan nanoparticles in (A) AIJ and (B) AGJ.

The drug-loading and entrapment efficiency of TQ in the powdered TQ-NC complex were 9.89 and 98.1%, respectively. The cumulative release profiles of TQ from a physical mixture of TQ and chitosan and TQ-NC nanoparticles in artificial intestinal juice and artificial gastric juice are shown in Fig. 2. In artificial intestinal and gastric juices, 100% of TQ detected in the physical mixture within the first 7 min. However, the release of TQ from TQ-NC nanoparticles was slow and gradual in both in the artificial intestinal and gastric juices. In artificial gastric juice the release of TQ from TQ-NC nanoparticles exhibited a “burst effect”, reaching 100 % released only after 1600 min. In artificial intestinal juice the release of TQ was slow and sustained with 100% release of TQ from TQ-NC nanoparticles after approximately 6000 min.

#### 3.1.8 Release kinetics of thymoquinone TQ from nanocomposites

**Fig. 3:**
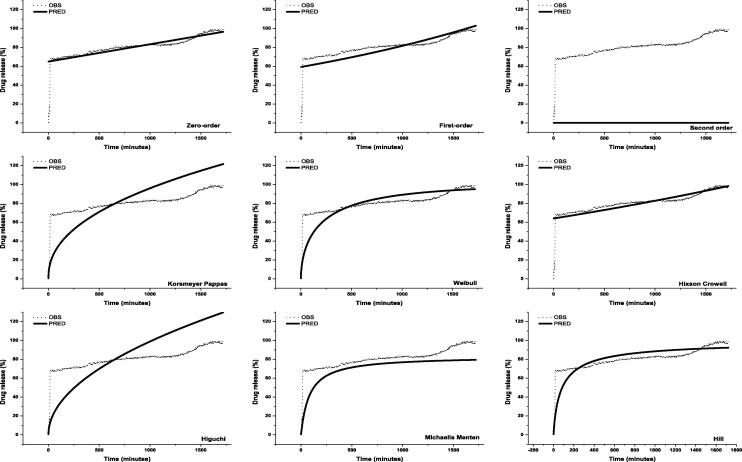
Kinetic models of thymoquinone (TQ) release from TQ-loaded chitosan nanocomposite in artificial gastric juice. OBS = observed value, PRED = predicted value.

**Fig. 4:**
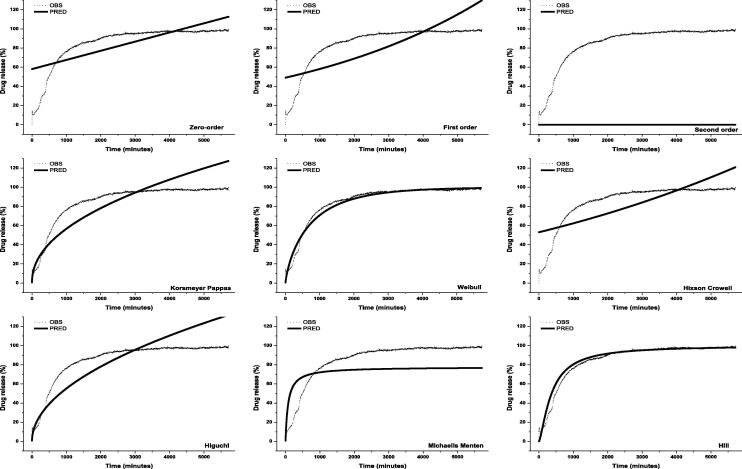
Kinetic models of thymoquinone (TQ) release from TQ-loaded chitosan nanocomposite in artificial intestinal juice. OBS = observed value, PRED = predicted value.

The TQ release profile from the TQ-NC were fitted to kinetic equations (Fig. 3, Fig. 4). The highest coefficient of determination and empirical R^2^, lowest Akaike information criterion, Bayesian information criterion Schwarz criterion and root-mean-squared error produced the best model (Table 2, Table 3). The analysis showed that the zero-order and Weibull models best describe the release kinetics of TQ from TQ-NC in artificial gastric and intestinal juice, respectively. With the best models, the release of TQ in artificial gastric and intestinal juices showed a coefficient of determination of 0.72527 and 0.95250, respectively. The root-mean-squared error of zero-order and Weibull models in artificial gastric and intestinal juices was 5.6122 and 3.1986, respectively.

**Table 2:** Data-fitting for thymoquinone (TQ) release from TQ-loaded chitosan nanocomposite in artificial gastric juice.

Model	Slope		Intercept	R^2^	R^2^_emp_	RMSE	AIC	BIC	*k*
Zero-order	0.018333		64.998252	0.72527	0.72527	5.6122	18787.7	18798.6	–
First-order	0.000321		4.082294	0.05030	−50.91969	6.8783	19488.7	19499.6	–
Second-order	−20198.946171		23195123.197877	0.00174	−1174207991595.52000	81.4896	28007.5	28018.4	–
Korsmeyer-Peppas	0.436761		1.547313	0.55849	−1145.51714	18.8744	22967.2	22978.1	4.699
Weibull	0.567357		−3.124704	0.70509	−2077.48988	9.9435	20758.7	20769.6	22.753
Hixson-Crowell	0.000358		3.999976	0.41713	−50.98426	5.7173	18851.6	18862.5	3.579E-04
Higuchi	0.500000		1.140254	−3.48387	−3.48387	22.6730	23599.1	23610.0	3.128
Michaelis-Menten	1.00000		0.0120035	1.00000	−7388.57273	12.5682	21565.9	21576.8	83.309
Hill	0.827436		−3.675446	0.43196	−4851.50013	8.3031	20137.4	20148.3	39.466

	**MTD**	330.607							
	**DE**	80.8010							
	**No of timepoints**	1723

**Table 3:** Data-fitting for thymoquinone (TQ) release from TQ-loaded chitosan nanocomposite in artificial intestinal juice.

Model	Slope		Intercept	R^2^	R^2^_emp_	RMSE	AIC	BIC	*k*
Zero-order	0.009557		58.080231	0.56309	0.98530	13.8880	79516.2	79529.5	-
First-order	0.000170		3.893840	0.248789	0.27243	17.9217	82430.7	82444.0	-
Second-order	−1836.719329		6997288.415651	0.00052	−16.51437	87.9307	100610.6	100623.9	-
Korsmeyer-Peppas	0.465114		0.824011	0.78865	0.48868	15.0242	80415.0	80428.3	2.280
Weibull	0.785649		−5.219769	0.95250	0.9**8**682	3.1986	62733.6	62746.9	184.891
Hixson-Crowell	0.000208		3.757296	0.45555	0.45107	15.5669	80820.6	80833.9	2.079E-04
Higuchi	0.500000		0.557263	0.34841	0.34841	16.9602	81800.4	81813.7	1.746
Michaelis-Menten	1.000000		0.012873	1.00000	0.16833	19.1610	83195.0	83208.3	77.680
Hill	1.415696		−8.368666	0.848949	0.96388	3.9932	65269.4	65282.7	4309.883

	**MTD**	834.827							
	**DE**	85.3898							
	**No of timepoints**	5715							

### 3.2 Anti-inflammatory activity

#### 3.2.1 Cytotoxicity

**Fig. 5:**
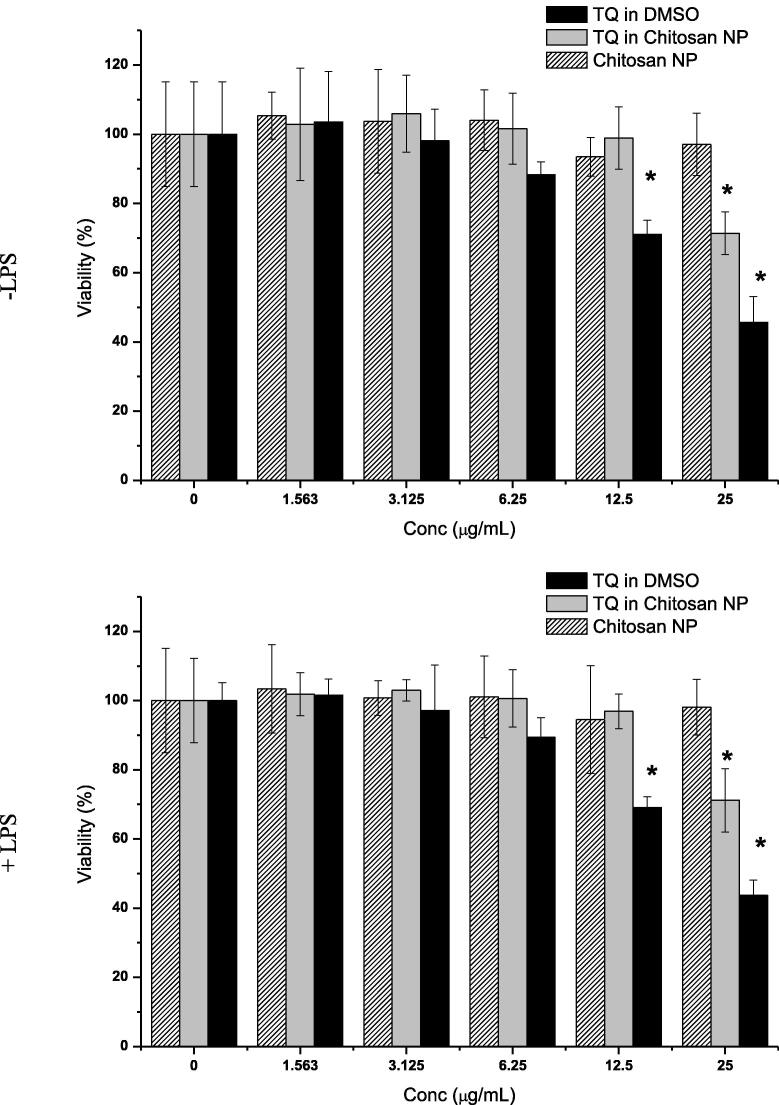
Viability of RAW 264.7 cells treated with chitosan nanoparticle, thymoquinone (TQ) dissolved in DMSO or encapsulated within chitosan nanoparticles after 24 h determined by the MTT assay. The cells were treated with serial concentrations of above mentioned compounds for 24 h then challenged with lipopolysaccharide LPS (100 ng/ml) for two hours. Values are mean ± SD (n = 3 wells/treatment). *Mean significantly different from mean of non-treated LPS-stimulated cells at P < 0.05.

The effects of chitosan nanoparticle, TQ dissolved in DMSO or encapsulated within chitosan nanoparticles on the RAW264.7 cells are shown in Fig. 5. None of these compounds significantly (P > 0.05) inhibits cell growth at concentrations of 1.6–6.3 μg/mL. Slight cytotoxicity was detected with pure TQ at 6.3 μg/ml after 24 h treatment. However, the challenging with LPS (100 ng/ml) showed no notable differences in cells viability with respect to those treated with compounds alone. The TQ at 2 μg/ml did not affect the viability of the cells. Thus the lowering effects of our compounds on proinflammatory markers are not attributed to inhibition in cells growth.

#### 3.2.2 Interleukin-6

**Fig. 6:**
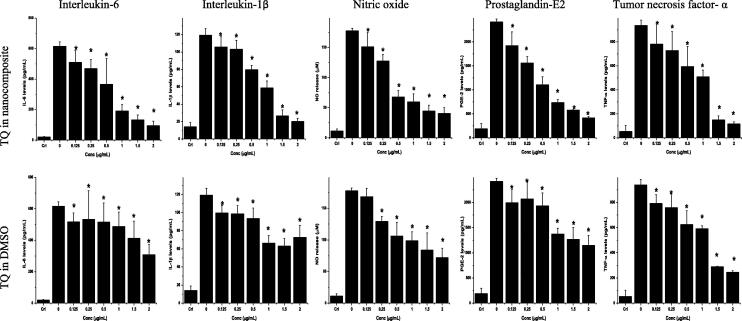
Levels of Interleukin-6 (IL-6), Interleukin-1β (IL-1β), Nitric oxide (NO), Prostaglandin-E2 (PGE-2) and Tumor necrosis factor-α (TNF-α) produced by lipopolysaccharide (LPS)-stimulated RAW 264.7 cells treated with thymoquinone (TQ) dissolved in DMSO or encapsulated within chitosan nanoparticles. Values are mean ± SD. *Mean significantly different from mean of non-treated LPS-stimulated cells at P < 0.05. Crl = negative control (non-LPS-stimulated RAW 264.7 cells).

To investigate the best concentration by which TQ-loaded chitosan nanoparticles could protect against inflammation, we applied different doses for anti-inflammatory assay. The concentrations of IL-6 produced by LPS-stimulated RAW 264.7 cells increased from 19.9 ± 3.4 to 615.2 ± 28.3 pg/mL after two hours (Fig. 6). IL-6 production by cells treated with 2.0 μg/mL TQ encapsulated in water soluble mushroom nanochitosan reached the minimal levels of 95.8 ± 28.4 pg/mL (Fig. 6). TQ encapsulated in chitosan at 1.0, 1.5, and 2.0 μg/mL caused lower production of IL-6 by 39.2, 32.2, and 31.0%, respectively, in comparison to cells treated with the same concentrations of TQ dissolved in DMSO (Fig. 6). Chitosan nanoparticle (from 1.25 to 20 μg/mL) did not exhibit any change in IL-6 levels when compared to untreated LPS-stimulated RAW 264.7 cells. The IC_50_ values of TQ dissolved in DMSO and complexed with chitosan for the production of IL-6 by the RAW 264.7 cells were 2.000 and 0.649 μg/mL, respectively.

#### 3.2.3 Interleukin-1β

The IL-1β productions by LPS-stimulated RAW 264.7 cells treated with TQ dissolved in DMSO or encapsulated within nanoparticles are represented in Fig. 6. TQ encapsulated within nanoparticles had greater concentration-dependent inhibitory effect on IL-1β production by LPS-stimulated RAW 264.7 cells than TQ dissolved in DMSO. The inhibitory effect of TQ-NC nanoparticles was greater with increase in treatment concentrations. The IC_50_ value of TQ incorporated in nanochitosan for the production of IL-1β by the RAW 264.7 cells was 1.009 μg/mL.

#### 3.2.4 Nitric oxide

Resveratrol (5 μg/mL) inhibited LPS-stimulated RAW 264.7 cells nitric oxide release by 49.2% lower than that by the non-resveratrol-treated LPS-stimulated RAW 264.7 macrophages. TQ dissolved in DMSO or encapsulated within nanoparticles concentration-dependently decreased NO production by the LPS-stimulated RAW 264.7 cells (Fig. 6), with the greatest (P < 0.05) reduction in production at the highest concentration of 2.0 μg/mL used in the study. Inhibitory effects were obvious at concentrations which significantly (P < 0.05) reduced nitrite production by 27.2% and 28.2% (at 2.5 TQ) and 40.2 and 61.8 % (at 5 μg/mL TQ), respectively as compared to the nitrite production by untreated LPS-stimulated macrophages. Chitosan nanoparticle (from 1.25 to 20 μg/mL) did not exhibit any change when compared to untreated LPS-stimulated RAW 264.7 cells. IC_50_ values of nitric oxide release for TQ dissolved in DMSO or complexed with chitosan were 1.342 and 0.435 μg/mL, respectively.

#### 3.2.5 Prostaglandin E2

PGE-2 production increased following LPS exposure (2419 ± 59 pg/mL), whereas in the absence of LPS, PGE-2 level was (190 ± 109 pg/mL) which is close to baseline or were almost undetectable (Fig. 6). Resveratrol reduced LPS-stimulated RAW 264.7 cells PGE2 production by 87% lower than that by the non-resveratrol-treated LPS-stimulated RAW 264.7 cells. TQ dissolved in DMSO or encapsulated within nanoparticles, especially at the highest concentrations used, markedly inhibited PGE2 production by the LPS-stimulated RAW 264.7 cells (Fig. 6). Exposure to TQ incorporated in nanochitosan lead to significant decrease (p < 0.05) in PGE-2 levels as a compared to nontreated cells by 82.8%, 76.1% and 69.5% from LPS-stimulated cells (2419.25 ± 58.98 pg/mL) to: (415.47 ± 27.88 pg/mL), (578.91 ± 9.25 pg/mL) and (736.90 ± 57.75 pg/mL), respectively. The effect of TQ encapsulated within nanochitosan is in concentration-dependent with decreasing PGE-2 production with increasing treatment concentrations. The IC_50_ values for TQ dissolved in DMSO or encapsulated within nanoparticles on inhibition of PGE-2 production were 1.757 and 0.461 μg/mL, respectively.

#### 3.2.6 Tumor necrosis factor-α

TQ dissolved in DMSO or encapsulated within nanoparticles at all concentrations had similar effect on the production of TNF-α by LPS-stimulated RAW 264.7 cells, although at high concentrations of 1.5 and 2.0 μg/mL, TQ incorporated in nanochitosan produced slightly more potent effect than TQ dissolved in DMSO (Fig. 6). The IC_50_values of TQ dissolved in DMSO or encapsulated within nanoparticles on TNF-α production by RAW 264.7 cells were 1.181 and 1.057 μg/mL, respectively.

## 4 Discussion

Water-soluble mushroom chitosan is a biopolymer that has great potential as a carrier for TQ. The loading of TQ in chitosan is simple, fast, efficient, and inexpensive. In this study we synthesized, characterized, and determined the anti-inflammatory properties of TQ-NC nanoparticles. The TQ-NC nanoparticles contained approximately 10% (w/w) thymoquinone. The XRD analysis showed that TQ was fully encapsulated in TQ-NC nanoparticles. The nanoparticles were stable and possessed the required functional groups to produce beneficial biological effects. The TEM showed that the TQ-NC nanoparticles were very small in size and diameter, while the DSC analysis showed that loading of TQ into chitosan did not affect the thermal stability of the TQ-NC complex.

Thermogravimetry is one of the most important thermal analytical technique for assessment of polymeric systems. The technique can be used to determine gain or loss in nanoparticle mass as a function of time and temperature. It is also a means of determining the range of temperature at which nanoparticles are chemically stable and reactions they undergo, which include oxidation, combustion, and dehydration (Al-Qubaisi et al., 2019). Chitosan, with the presence two polar groups, hydroxyl and amine, has high affinity for water molecules, thus, easily hydrated. However, the acquisition of water molecules can cause disorder in the structure of the polysaccharide. During the formulation of TQ-NC nanoparticles, the chitosan can lose their water contents, resulting in decrease in mass. The water loss from the TQ-NC nanoparticles was detected in the thermogravimetric analyses.

The utility of TQ-NC nanoparticles as a potential therapeutic compound lies on the release characteristics of TQ, whether sustained or burst. Ideally, nanoparticulate drug carriers meant for oral or parenteral applications should exhibit sustained-release characteristics for full therapeutic potential. However, in the study, it appears that the TQ-NC nanoparticles are not suitable for oral application. This is due to the fact that the release of TQ from TQ-NC nanoparticles is pH sensitive. The pH of human gastric juice is between 1.5 and 3.5 while for the intestinal juice it is between 6.0 and 7.4 (Al-Qubaisi et al., 2019). At pH 6.8, TQ release from TQ-NC nanoparticles was sustained over a period of 100 h. However, at pH 1.2 the nanoparticles exhibited rapid burst release of their TQ load that lasted approximately 26 h. Thus, in the gastric juice, the TQ-NC nanoparticles would rapidly release TQ, which compromises their utility as a systemic carrier for orally administered therapeutic compounds. The release of TQ from TQ-NC nanoparticles at pH 6.8 followed the Weibull kinetic release model and this is primarily the result of nanoparticle dissolution rather than diffusion of TQ from the nanoparticles (Al-Qubaisi et al., 2019). The sustained-release feature of TQ-NC nanoparticles at pH 6.8 has important biological implications. The intracellular pH of normal cells is approximately 7.2 (Al-Abboodi et al., 2021). Thus, in the intracellular environment, the TQ-NC nanoparticles will release TQ slowly with prolonged biological effects.

The LPS-induced RAW264.7 macrophage is a classical model for the *in vitro* assessment of the anti-inflammatory activities (Park et al., 2005, Rhule et al., 2006, Shin et al., 2008, Cheng et al., 2012, Xu et al., 2014). In our study, TQ-NC nanoparticles concentration-dependently inhibited production of NO, PGE2, IL-6, TNF-α, and IL-1β by these cells. TNF-α and IL-1β could induce release of secondary cytokines, chemokines and non-cytokine mediators, which play distinctive roles in inflammation (Serhan et al., 2010). TNF-α, IL-1β and IL-6 are associated with clinical signs of inflammation and can induce fever (Kozak et al., 1998, Dicuonzo et al., 2003). NO and PGE2 are good biomarkers for inflammation (Vane et al., 1994, Shen et al., 2002, Yoon et al., 2010). NO is anti-inflammatory under normal physiological conditions and pro-inflammatory when there is excessive production under abnormal conditions (Sharma et al., 2007). NO in excess also modulates several pathological changes associated with inflammation. PGE2, on the other hand, is responsible for vasodilatation, plasma leakage, and perception of pain during inflammation, effects that facilitates healing (Williams, 1983, Markowitz et al., 1987, Waeber and Moskowitz, 2005, Birklein and Schmelz, 2008). Thus, based on their ability to reduce macrophage cytokine production, the TQ-NC nanoparticles clearly have potential to be developed into an efficacious anti-inflammatory compound. The inhibitory effects of TQ-NC on NO and PGE2 productions also prevent damage to tissues from the cytotoxic and tissue damaging effects of these cytokines. The inhibition of NO and of PGE2 production by macrophages is suggested to occur through regulation of inducible nitric oxide synthase and cyclooxygenase-2, respectively (Nussler and Billiar, 1993, Vane et al., 1994).

Chitosan and TQ each on its own has anti-inflammatory properties (Kalamegam et al., 2020). The complexation of TQ with chitosan had provided the TQ-NC nanoparticles with enhanced anti-inflammatory properties. The TQ-NC nanoparticles are highly water-soluble and will rapidly be adsorbed at body surfaces, exhibit prolonged transit time in circulation, show improved tissue targeting and deposition, and control release of the TQ load, which are features of an efficient drug delivery system.

## 5 Conclusion

TQ can be efficiently and effectively incorporated onto chitosan nanoparticle. The successful complexation of TQ with chitosan produced TQ-NC nanoparticles with sustained release properties of their TQ loads, especially at pH 6.8. TQ-NC modulates production of the inflammatory mediators by the macrophage RAW 264.7 cells. In conclusion, TQ-NC nanoparticles have great potential to be developed as an efficacious parenteral anti-inflammatory compound.

## Declaration of Competing Interest

The authors declare that they have no known competing financial interests or personal relationships that could have appeared to influence the work reported in this paper.
